# The Impact of Ageing on Episodic Memory Retrieval: How Valence Influences Neural Functional Connectivity

**DOI:** 10.3390/neurosci5040040

**Published:** 2024-11-11

**Authors:** Marianna Constantinou, Anna Pecchinenda, Hana Burianová, Ala Yankouskaya

**Affiliations:** 1 Department of Psychology, Bournemouth University, Bournemouth BH12 5BB, UK; mconstantinou@bournemouth.ac.uk; 2 Department of Psychology, Sapienza University of Rome, 00185 Rome, Italy; anna.pecchinenda@uniroma1.it; 3 School of Psychology, Swansea University, Swansea SA2 8PQ, UK; hana.burianova@centrumdohody.com

**Keywords:** healthy ageing, episodic memory, fMRI, valence, arousal

## Abstract

Age-related decline in episodic memory is often linked to structural and functional changes in the brain. Here, we investigated how these alterations might affect functional connectivity during memory retrieval following exposure to emotional stimuli. Using functional magnetic resonance imaging (fMRI), participants viewed images with varying emotional valences (positive, negative, and neutral) followed by unrelated non-arousing videos and were then asked to retrieve an episodic detail from the previously shown video. We conducted Multivariate Pattern Analysis (MVPA) to identify regions with divergent responses between age groups, which then served as seeds in Seed-Based Connectivity (SBC) analyses. The results revealed an age-related decline in behavioural performance following exposure to negative stimuli but preserved performance following positive stimuli. Young adults exhibited increased functional connectivity following negative valence. Conversely, old adults displayed increased connectivity more scarcely, and only following positive valence. These findings point to an adaptive response of the impact of emotions on task performance that depends on neural adaptations related to ageing. This suggests that age-related changes in functional connectivity might underlie how emotions influence memory, highlighting the need to tailor memory support strategies in older adulthood.

## 1. Introduction

Episodic memory, which allows the conscious retrieval of personal experiences and specific events, including their contextual details [1], tends to deteriorate as people age [2,3,4,5,6,7,8]. This age-related decline is associated with decreased performance on tasks that assess the retrieval of events encoded in the laboratory (episodic laboratory retrieval [9]), with old participants exhibiting poorer performance than young participants [5,6,10,11]. This underscores the challenges faced by the elderly in retrieving specific events accurately. In healthy ageing, the retrieved output tends to be more generalised and lacks specificity [12,13,14]. This may be due to a difficulty in updating bindings between items and their contextual details [15]. Additionally, healthy ageing is associated with reduced awareness and monitoring of episodic memory processes [16,17] which may further contribute to the observed decline in performance. Greater variability in response speed during retrieval is also observed in old compared to young adults [18,19], correlating with poorer episodic memory performance [20].

The medial temporal lobe (MTL), which is crucial for encoding and retrieval of episodic memory, shows structural changes as it ages [21,22]. Specifically, the hippocampus exhibits significant age-related atrophy, and longitudinal studies have linked its shrinkage to declines in episodic memory [23,24,25]. Additionally, reduced hippocampal activity is consistently associated with worse episodic memory [26,27,28]. Ageing is also linked to increased activation in the prefrontal cortex (PFC) during memory retrieval [27,29,30,31,32,33], with older adults who perform better on memory tasks exhibiting increased bilateral activity in the prefrontal regions compared to those who perform poorly [29]. This suggests that increased engagement of bilateral prefrontal regions enhances memory performance. It is plausible that increases in PFC activity, along with its enhanced coupling with other brain regions, may help address episodic-memory deficits associated with changes in the MTL [34]. This finding provides strong support for the compensation hypothesis in ageing [9,35]. Additionally, overactivation in old adults sometimes mirrors that of young adults but often occurs in the opposite hemisphere, a phenomenon known as hemispheric asymmetry reduction in old adults (HAROLD) [29]. However, the compensatory activation, as described by the compensation-related utilisation of neural circuits hypothesis (CRUNCH) has its limits. While beneficial for simple tasks, it becomes less effective as task complexity increases, leading to performance declines for more challenging tasks [36]. Recent findings show that stimulating the cerebellum can also improve episodic memory in old adults [37], indicating that it is equally important to consider how regions across the whole brain interact to adaptively respond to age-related episodic memory decline.

The retrieval of episodic memory is significantly affected by the emotional context of the stimulus to be retrieved, which is characterised by levels of arousal (i.e., high or low) and valence (i.e., ranging from negative to positive) [38] as indicated in [39,40,41,42]. Some studies report that highly arousing stimuli are retrieved more accurately and quickly [43,44,45,46]. This memory enhancement was further linked to the functional connectivity among different brain regions across the whole brain, which are part of different functional networks, including the dorsal attention network, default-mode network, and salience network [46]. This finding emphasises that arousal-dependent episodic memory is not limited to specific regions but also involves networks throughout the entire brain. Furthermore, it is established that high arousal not only influences the initial encoding of arousing stimuli but also impacts the encoding and retrieval of information presented before and after these stimuli [47,48,49]. Negative images, compared to neutral ones, lead to faster and more accurate responses, possibly due to an increase in attention to subsequently presented auditory stimuli [50]. However, evidence shows that negative stimuli hinder associative binding, weakening memory coherence for nearby neutral objects [51]. This finding offers a valuable insight into the dual role of negative valence in enhancing information binding within a shared context while impeding memory for nearby neutral objects. To our knowledge, there is no published work specifically exploring how functional connectivity influences the retrieval of nearby objects following arousal. While recent research demonstrates that arousal affects the brain on a holistic level, impacting multiple neural networks [46], the specific effect on connectivity related to the retrieval of information presented following arousal exposure is yet to be examined. Investigating this becomes even more crucial when considering ageing populations, as we know that age affects resting and task-based functional connectivity [52,53,54]. Including both young participants and old participants helps challenge the assumption that functional connectivity does not change with age and reveals how ageing may specifically influence the effects of arousal on episodic memory retrieval. This approach may identify targeted strategies to mitigate episodic memory decline in older adults.

Apart from stimulus arousal, valence also has the potential to affect age-related changes in episodic memory, as there is a positivity shift with ageing [55,56,57]. The enhancement may stem from an age-related decline in cognitive functions of brain areas that process emotional stimuli, potentially leading to a less efficient processing of specifically negative events [58]. This could result in a shift towards a more positive emotional focus, consistent with the positivity effect [59], which may represent an adaptive mechanism to optimise emotional well-being in the ageing brain. Emotion-related processes involve key regions across the entire brain—predominantly the prefrontal cortex [60,61,62,63], including the anterior cingulate cortex [64,65], and areas within the temporal lobe such as the amygdala and hippocampus [66,67]. Given the involvement of various regions across the entire brain, it is essential to consider the interactions among these regions to understand the changes associated with ageing. For instance, although the amygdala has been found to be active in both young adults and old adults, its functional connectivity is age-dependent, with old adults exhibiting increased functional connectivity between the amygdala and the ventral anterior cingulate cortex and decreased functional connectivity between the amygdala and the posterior visual brain regions [68]. Such findings give support to the Posterior–Anterior Shift in Ageing (PASA) theory, where old adults tend to show an increase in activity in anterior brain regions as well as a decrease in activity in posterior brain regions [69]. The decreased connectivity with these posterior regions in old adults may indicate reduced specialisation in perceptual processing areas, suggesting neural dedifferentiation, a pattern of broader neural engagement that can contribute to a cognitive decline [70,71].

Based on the established evidence that episodic memory declines with age and is influenced by emotional content, this study aims to examine the neural mechanisms underlying these changes. Here, we aim to examine the functional connectivity of regions across the whole brain that are involved in episodic memory retrieval of events presented following arousal exposure in young adults and old adults. We use a data-driven approach, which moves beyond examining specific regions in isolation, focusing instead on the coupling between regions that are significantly activated during episodic memory retrieval. Our objectives are twofold: (i) to examine how functional connectivity between key regions involved in episodic retrieval changes with age, particularly in response to prior exposure to emotionally arousing stimuli in young adults and old adults; and (ii) to investigate valence-specific effects on episodic retrieval, focusing on how responses to positive versus negative stimuli differ across age groups. We hypothesise age differences in functional connectivity, as well as variations in how positive and negative valence influence these connectivity patterns. This study seeks to clarify the role of functional connectivity in understanding episodic memory changes in the old adult brain, providing empirical support for existing ageing theories.

## 2. Methods

### 2.1. Participants

The final sample size included twenty-six young adults (mean age = 24 years; SD = 3.6; 8 males) and twenty old adults (mean age = 69 years; SD = 5.4; 9 males). Participants had normal or corrected-to-normal vision and had no colour vision impairments, no neuropsychiatric disorders, no head trauma, and no history of taking cardiovascular-related medication. Exclusion criteria included metal body implants for MRI comparability. All participants had a minimum of a higher education diploma, and all old adults showed preserved cognitive function based on the Mini-Mental State Examination (MMSE; scores between 28 and 30, average score = 29.9, SD = 0.48) [72]. The study received ethical approval from the Psychology Department’s Ethics Committee at Bournemouth University.

### 2.2. Task Stimuli

#### 2.2.1. Images

A total of 48 colour photographs featuring animals, humans, and scenes were used as cues for the experimental conditions, based on their valence ratings. Of these, 24 images were selected from the Open Affective Standardized Image Set (OASIS) [73] database, featuring images with positive valence. The remaining 24 images were chosen from the Geneva Affective Picture Database (GAPED) [74], featuring images with negative valence.

The rationale behind using two datasets was twofold. First, the GAPED database contains fewer pictures of positive content than negative content, limiting the ability to pair specific categories and balance content across valences [75]. However, negative pictures from this database have been consistently used in previous studies for their ability to elicit negatively valenced emotions [76,77]. Second, the OASIS database provides an excellent set of images with a broad range of positive content. The use of negative images from the OASIS database may introduce unwanted confounding effects, such as distress from sexually explicit, violent, or traumatic content [73]. These confounding effects could potentially result in additional head movements during scanning sessions, decreasing the quality of functional connectivity data [78].

To address these limitations, we selected the positive images with the highest ratings from the OASIS database and the negative images with the highest ratings from the GAPED database. We then assessed whether the ratings of the selected subsets were significantly different, ensuring they effectively represented the different valences. Since the GAPED ratings use a 0–100 scale and the OASIS ratings use a 7-point Likert scale, we applied a normalisation procedure, scaling both ratings from 0 to 1, where 0 represented negative and 1 positive valence, and compared them using an independent-sample *t*-test [79]. The test indicated significant differences between these ratings (t(26.31) = 28.25, *p* < 0.001). There was no difference between arousal ratings for the positive and negative images we used in the present study (see details in Appendix A).

A black-and-white scrambled image was used to represent the neutral condition, lacking valence and high arousal effects. Scrambled images were used to avoid the ambiguity and potential misinterpretation associated with emotionally neutral images. Scrambled images reduce the risk of assigning unintended emotional valence, offering a more consistent neutral baseline across various contexts (faces, nature, and animals). This method aligns with previous studies on emotional valence, allowing accurate comparisons [80,81,82,83]. The stimuli were presented using PsychoPy (v2021.2.3) [84].

#### 2.2.2. Video

Twenty-four videos were selected from the ActivityNet database [85] and trimmed to last 7 s each, aiming to strike a balance between providing participants enough context to answer follow-up questions and, at the same time, having enough time for multiple trials. ActivityNet provides an extensive collection of videos that capture a variety of everyday human activities. The chosen videos all featured simple, day-to-day activities without any emotionally charged content, including scenes of individuals engaging in task such as sports, music, cleaning, and cooking. Each video was presented three times. To ensure a comparison across different emotional valence conditions, each video was paired with different images in each presentation, with each video being associated with a positive, negative, and neutral condition once each. This pairing was randomised, and so not all subjects viewed the same videos with the same images.

### 2.3. Experimental Design

A 2 × 3 mixed-subjects design was used with Age as the between-subjects factor with two levels (young adults and old adults) and Condition as the within-subjects factor with three levels (negative valence, positive valence, and neutral valence). The dependent variables included changes in the BOLD response [86], response times (RTs), and accuracy.

### 2.4. Task and Experimental Procedure

**Figure 1:**
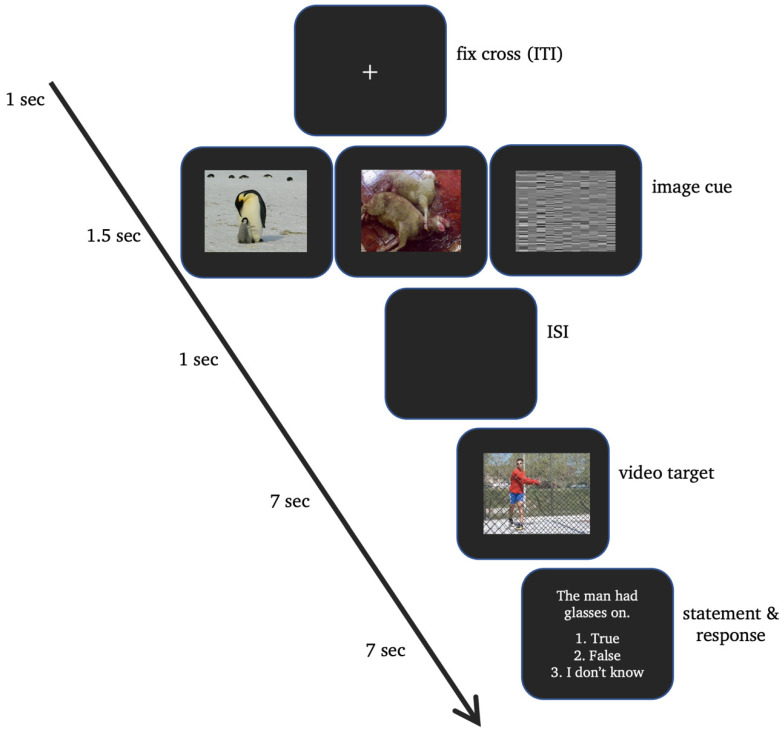
Task schematic. Each trial began with an image cue (1.5 s) that was positive (left), negative (middle), or neutral (right) in valence, followed by an ISI (1 s). A video then was displayed (7 s), followed by a statement and a response period (7 s). Participants indicated their responses by pressing “True”, “False”, or “I don’t know”. The trial continued with an ITI (1 s) represented by a fixation cross.

Once they signed informed consent, only the old adults took a cognitive pre-screening test using the MMSE [72]. Participants were informed that images would appear briefly on the screen, followed by a video. They were instructed to pay close attention to the video, as they would need to respond to statements about the video afterward. All participants practiced a single trial of the task outside the brain scanner. Inside the scanner, participants were instructed how to use the compatible response box, with the index finger for “True”, middle finger for “False” and ring finger for “I don’t know” responses. The experiment commenced with a 7-min structural scan, then moved to the main task, consisting of 72 trials across three functional runs, presented in a randomised order. Each trial displayed an image, followed by a video and a statement, giving participants 7 s to respond, with only the initial response recorded. Upon completion, the purpose of the study was disclosed to the participants. For the task schematic, refer to Figure 1.

### 2.5. fMRI Data Acquisition

Brain images were collected using a 3 T Siemens Magnetom Lumina scanner. T1-weighted anatomical MRI was obtained using an MP2RAGE sequence with 176 sagittal slices, TR = 4000 ms, TE = 2.98 ms, FOV = 256 mm, flip angle = 4°, and voxel size = 1 mm^3^.

For functional imaging, blood oxygenation level-dependent (BOLD) contrast whole-brain images were captured using a T2-weighted gradient-echo Echo-Planar Imaging (EPI) sequence with a 32-channel head coil. The acquisition parameters included a TR = 3000 ms, TE = 30 ms, matrix size = 64 × 64, in-plane resolution 2.5 × 2.5 mm, slice thickness = 2.5 mm, field of view (FOV) = 190 mm, and a flip angle = 90°. A total of 420 volumes with 46 axial slices were measured in interleaved slice order and positioned along a line connecting the anterior and posterior commissures (AC–PC orientation). An automated high-order shimming technique was used to maximise magnetic field homogeneity.

### 2.6. Behavioural Data Analysis and Mixed Modelling

Response times exceeding 2.5 SDs from each participant’s mean were deemed outliers and excluded along with their accuracy data. Response times were normalised into z-scores to account for age-related variations in speed and processing [87]. Linear mixed modelling was used to examine Age and Valence effects with fixed effects including Age, Valence, Video Repetition, and the interaction between Age and Valence. The inclusion of Video Repetition as a fixed effect ensures that any potential bias introduced by the repeated presentation of video stimuli is appropriately accounted for in the analysis. By treating Video Repetition as a fixed effect, the statistical model adjusts the estimates for each level of this variable to account for systematic differences in responses that are attributable to having seen the videos multiple times [88]. Random effects in the model included Participant and Video Stimulus. Accuracy and response times were assessed using generalised and linear mixed modelling, respectively, using RStudio (v2023.03.0) [89]. The use of mixed models is particularly advantageous in this study, as they accommodate the unbalanced data arising from unequal group sizes, ensuring robust estimation of effects across groups [88].

### 2.7. fMRI Data Preprocessing and Denoising

SPM12 (www.fil.ion.ucl.ac.uk/spm) [90] and the CONN toolbox (version 21a; http://www.nitrc.org/projects/conn) [91] were used to preprocess the functional neuroimaging data in Matlab R2022a [92].

The preprocessing of the functional data included realignment (correction for head motion-induced intervolume displacement), unwarping, slice-timing correction (differences in acquisition time), and segmentation and normalisation to the Montreal Neurological Institute (MNI) space using the default TPM MNI template and the unified normalisation–segmentation procedure. After the images were aligned to the MNI space by affine alignment, they were resliced to 2 × 2 × 2 mm and smoothed with an isotropic 6 mm FWHM Gaussian kernel. Default high-pass temporal filtering (1/128 Hz cut-off) in SPM12 was applied to remove low-frequency noise and signal drifts from each voxel’s fMRI time course. Then, 1st-level modelling was conducted, and these models were entered into the CONN toolbox, which was used to perform denoising.

Utilising CONN’s default denoising pipeline, we aimed to further mitigate the influence of residual noise and non-neural variability in the BOLD signal, ensuring the robustness of our functional connectivity analyses. The pipeline combines linear regression to remove potential confounding effects from the BOLD signal and temporal band-pass filtering. Through Ordinary Least Squares (OLS) regression, identified potential confounding effects, including noise components from cerebral white matter and cerebrospinal areas [90] using an anatomical component-based noise correction procedure (aCompCor), estimated subject-motion parameters [93], and task effects [91] were individually projected to the sub-space orthogonal to the BOLD signal timeseries for each voxel and subject. Subsequently, temporal frequencies below 0.008 HZ and above 0.09 Hz were removed from the BOLD signal to focus on slow-frequency fluctuations and minimize the influence of noise, enhancing the reliability of the functional connectivity analyses.

### 2.8. fMRI Data Analysis

#### 2.8.1. Multivariate Pattern Analysis (MVPA)

To investigate age-related differences in brain functional connectivity across our conditions, we employed Multivariate Pattern Analysis (MVPA) using the CONN toolbox. This data-driven technique aimed to identify regions of interest based on connectivity patterns, which would later serve as seeds for further analyses, e.g., [94,95].

MVPA involves creating eigenpattern score maps that represent how each voxel’s connectivity with the entire brain varies across subjects and the three conditions. This is established by conducting a Singular Value Decomposition for each voxel, capturing how connectivity patterns between that voxel and the rest of the brain differ across groups and conditions [96]. The outcome defines arbitrary diverse connectivity patterns, facilitating the examination of age-related connectivity differences through secondary multivariate analysis on the generated eigenpattern maps.

To define data-driven seeds, global connectivity was calculated for each condition separately, in young adults and old adults. Principal Component Analysis (PCA) was applied to reduce the data, with two factors selected to account for variations. The decision of how many components to include in MVPA second-level analysis depended on our data. When group differences in connectivity are pronounced, they are typically captured by the first components, while weaker differences are captured by higher-order components. This decision was guided by the practice of selecting principal components in a range of 10–20% of the number of subjects per group, which, herein, falls between 2 and 3 factors [97,98]. While a consensus is lacking on the ideal sample/principal component ratio for PCA, a prevailing trend in research leans towards higher ratios, such as 20:1 [98,99].

#### 2.8.2. Seed-Based Connectivity (SBC) Analyses

Following the MVPA analysis, we proceeded with a subsequent SBC analysis that capitalised on a data-driven approach. This involved leveraging the clusters derived from the group MVPA results as the seeds of interest for the first SBC analysis. The ROIs were entered into CONN and fed into the standard preprocessing and denoising pipelines to ensure consistency before conducting the SBC analysis. To begin, we exported connectivity maps for each contrast (negative vs. neutral; positive vs. neutral; negative vs. positive) and clusters that demonstrated significance below the 0.005 (FWE-corrected) threshold were then separated from the aggregated contrast maps. To gain a more comprehensive understanding, these seeds were subsequently examined in terms of their functional connectivity with the entire brain. This approach aimed to elucidate how these specific data-driven regions of interest, identified as exhibiting consistent age-related differences, interacted with other brain regions during memory retrieval following the different valence conditions. By exploring the broader functional connectivity patterns associated with these seeds, we aimed to uncover the underlying changes in functional connectivity shaping age-related distinctions in episodic memory retrieval.

#### 2.8.3. Semipartial Correlations

Finally, to further discern the functional connectivity differences highlighted by the MVPA, we conducted an SBC analysis employing semipartial correlation techniques. This step was crucial for distinguishing whether the variations in connectivity patterns identified could be attributed to the unique influence of individual seeds, or whether they were a result of interdependencies among multiple seed regions. Semipartial correlation analyses allow the isolation of the unique impact of each seed region on the observed whole-brain connectivity patterns, while controlling for potential confounding effects stemming from interactions with other seeds. In other words, by applying semipartial correlation, we aimed to ensure that the connectivity patterns identified are directly linked to the specific seed under consideration, as opposed to being indirectly influenced by its relations with other seeds. This approach facilitates a more specific comprehension of how age-related distinctions in connectivity between each seed and the rest of the brain contribute to functional connectivity changes.

## 3. Results

### 3.1. Behavioural Results

**Figure 2:**
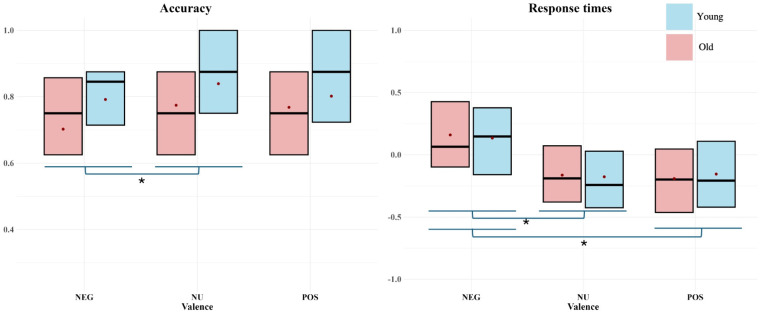
Behavioural results: Box plots showing accuracy (**left**) and RTs (**right**) by age group and valence. Each box represents the interquartile range, with the lines representing the median of each condition (NEG = negative; NU = neutral; POS = positive), and the dots showing the mean of each condition. Asterisk (*) denotes statistical significance (*p* < 0.005).

In summary, the behavioural findings shown in Figure 2, indicate an effect of Age on accuracy performance, revealing that young adults performed significantly better compared to old adults. Moreover, negative valence impacted accuracy across age groups, with both young adults and old adults showing reduced accuracy following negative compared to neutral valence. Moreover, the findings do not show a significant influence of ageing on response time performance. Both young adults and old adults were significantly slower in the negative valence condition compared to the neutral and positive valence conditions (for details, see Appendix B).

### 3.2. fMRI Results

#### 3.2.1. MVPA

**Figure 3:**
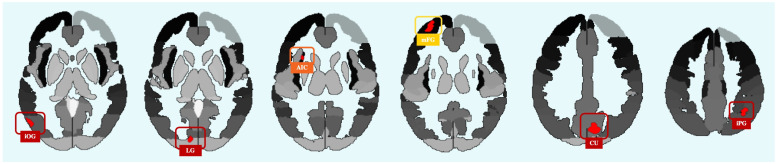
MVPA results. Significant voxel-to-voxel differences in functional connectivity between young adults and old adults across six regions. The regions in red (l iOG, l LG, r CU, r iPG) indicate variations during exposure to negative versus neutral valence, the region in orange (l AIC) denotes variation between highlights variation under positive versus neutral conditions, and the region in yellow (l mFG) denotes variation between negative and positive conditions. Abbreviations: AIC = anterior insular cortex; CU = cuneus; iOG = inferior occipital gyrus; iPG = inferior parietal gyrus; LG = lingual gyrus; mFG = middle frontal gyrus.

Comparisons between young adults and old adults revealed variations in functional connectivity across the entire brain in six seeds. We focused on regions with significant connectivity differences, setting our cut-off at 70 voxels, which aligns with previous MVPA studies with a threshold between 50 and 100 voxels, e.g., [97,100]. Four of these regions displayed significant functional connectivity variations during negative compared to neutral stimulus exposure. Notably, the right cuneus, left lingual gyrus, left inferior occipital gyrus, and right inferior parietal gyrus demonstrated distinctive patterns in response to negative valence. Additionally, contrasting the positive condition with the neutral condition highlighted the involvement of the left anterior insula, indicating unique neural processing associated with positive valence. Furthermore, the comparison between negative and positive conditions revealed differences in the left middle frontal gyrus. Refer to Figure 3 and Table 1 for a depiction of all seed regions (significant at *p*-FWE > 0.005) derived from the MVPA analysis.

This analysis identifies differences in functional connectivity between conditions but does not elucidate whether each seed showed increased or decreased connectivity. To address this, follow-up analyses were conducted using the MVPA results as seed regions for subsequent analyses.

#### 3.2.2. SBC

In a detailed examination of age-related differences in functional connectivity, we focused on the six seed regions identified through MVPA for exhibiting significant variations across the three conditions (negative, positive, and neutral). In this initial SBC analysis, we did not control for interdependencies among multiple seed regions to grasp an idea about all FC differences. The results of the first SBC using bivariate correlations can be found in Appendix C.

#### 3.2.3. SBC—Semipartial Correlations

Examining the data more closely and isolating the unique influence of each seed regions, we found that the left lingual gyrus and left inferior occipital gyrus which initially showed differences in functional connectivity, no longer displayed differences when controlled for using semipartial correlations.

First, we report age-related differences in functional connectivity without considering valence-related effects. Specifically, in young adults, compared to old adults, the right inferior parietal gyrus was functionally connected to the right precuneus and right lingual gyrus. Additionally, in young adults, the left anterior insula showed increased functional connectivity with the left precentral gyrus, the right superior medial frontal gyrus, and the left lingual and fusiform gyri. Finally, increased connectivity for the young adults was observed between the left middle frontal gyrus and the left paracentral lobule. On the other hand, in old adults, compared to young adults we report increased functional connectivity only between the right cuneus and the right precentral gyrus and between the left anterior insula and the left inferior occipital gyrus.

**Figure 4:**
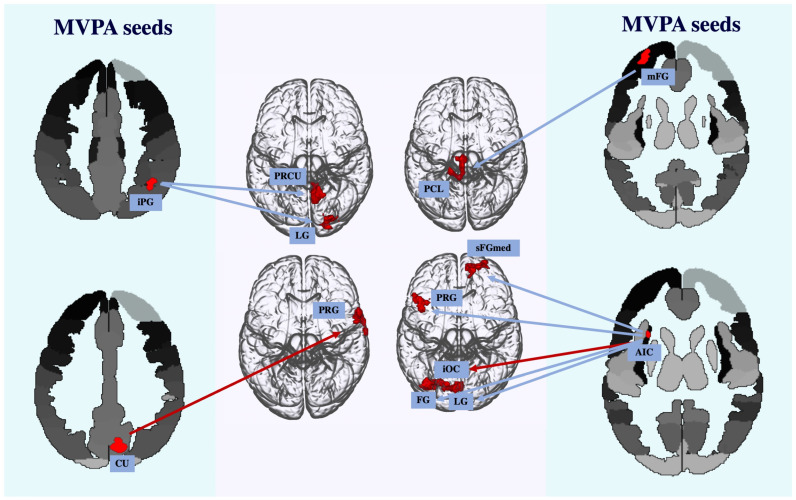
SBC results using semipartial correlations. Significant increases in functional connectivity in young adults compared to old adults (indicated via the blue arrow) and in old adults comparted to young adults (indicated via the red arrow) between the MVPA seed regions and the rest of the brain after controlling for interdependencies. Abbreviations: AIC = anterior insular cortex; CU = cuneus; FG = fusiform gyrus; iOC = inferior occipital gyrus; iPG = inferior parietal gyrus; LG = lingual gyrus; mFG = middle frontal gyrus; PCL = paracentral lobule; PRCU = precuneus; PRG = precentral gyrus; sFGmed = superior medial frontal gyrus.

**Figure A3:**
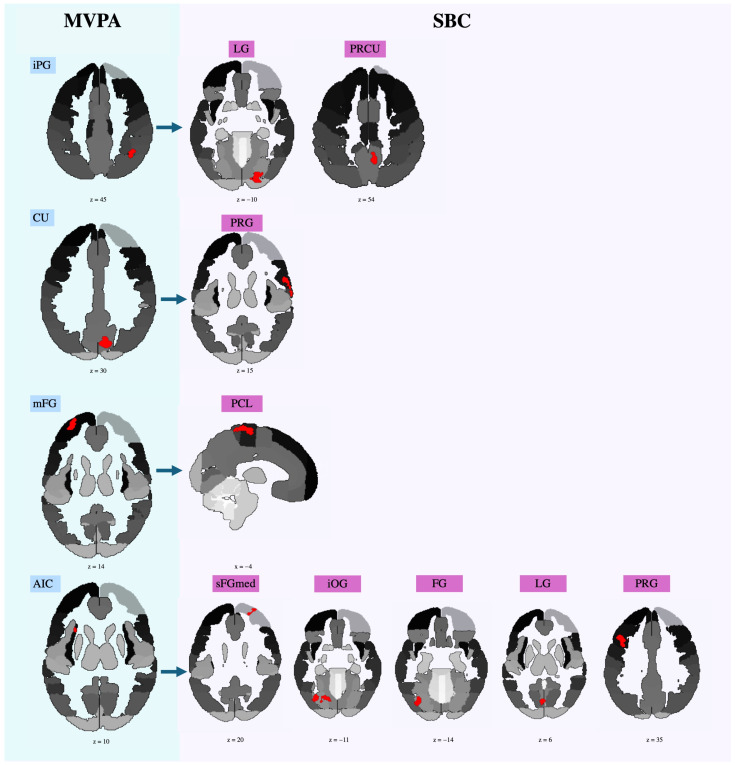
SBC results using semipartial correlations. Significant increases in functional connectivity between the MVPA seed regions and the rest of the brain, after controlling for interdependencies. Each MVPA finding is connected to the SBC result displayed on the same row. Each SBC result is displayed on a separate slice for clarity. Abbreviations: AIC = anterior insular cortex; CU = cuneus; FG = fusiform gyrus; iOC = inferior occipital gyrus; iPG = inferior parietal gyrus; LG = lingual gyrus; mFG = middle frontal gyrus; PCL = paracentral lobule; PRCU = precuneus; PRG = precentral gyrus; sFGmed = superior medial frontal gyrus; x coordinate = right/left; z coordinate = inferior/superior.

For valence differences in functional connectivity, we found that in old adults, increases in functional coupling were observed only for the positive compared to the neutral condition. However, in young adults, an increase in functional connectivity between the right inferior parietal gyrus and the right precuneus and between the left anterior insula and the left precentral gyrus was observed for the negative compared to the neutral condition. For the negative compared to the positive condition, increased functional connectivity was again found between the right inferior parietal gyrus and the right precuneus, between the left anterior insula and both the left lingual and left fusiform gyri, and between the left middle frontal gyrus and the left paracentral lobule. Finally, for the positive compared to neutral condition, increased connectivity was found following positive valence between the right inferior parietal gyrus and the right lingual gyrus and between the left anterior insula and the right superior medial frontal gyrus. Refer to Figure 4 and Table 2, which display all significant functional connections with clusters at *p*-FWE > 0.005 in young adults compared to old adults and old adults compared to young adults between conditions after controlling for interdependencies. Each SBC result is also displayed on a separate slice for clarity (see Figure A3 in Appendix D).

## 4. Discussion

The current study aimed to examine age-related differences in functional connectivity during episodic memory retrieval, focusing on how these differences were influenced by emotional valence. We hypothesised that old adults would exhibit distinct connectivity patterns compared to young adults, with variations influenced by the emotional valence participants were exposed to prior encoding. Using a data-driven approach, we identified key regions where connectivity differed markedly between young adults and old adults and examined how the functional coupling of these regions with the rest of the brain varied across age groups. We found that old adults had more difficulty retrieving episodic details, particularly after exposure to negative valence, which exacerbated the decline in memory retrieval of unrelated stimuli. Moreover, our analyses revealed distinct connectivity patterns in specific brain regions between young adults and old adults, highlighting age-related changes in neural communication. Further examination of these regions showed differences in their connectivity with other brain areas between the two groups, suggesting age-specific adaptations in functional connectivity. Additionally, we observed differential contributions of negative and positive valence to these connectivity patterns, underscoring the varying impact of emotional content on memory processes and ageing.

The behavioural results show that ageing negatively affects episodic memory retrieval, particularly accuracy performance, with young adults performing better than old adults, consistently aligning with previous studies [2,3,6,8]. This decline could be attributed to age-related cognitive decline due to structural and functional neural changes [23,24,25,26,28]. Furthermore, the impact of negative stimuli on accuracy in both young and old adults suggests that negative valence can continue to influence encoding of stimuli which are presented immediately following arousal exposure. This aligns with research indicating the strong impact of highly arousing negative stimuli [43,44] and with evidence supporting their disruptive effect on associative binding in young adults [51]. Our results did not reveal a significant age-related difference in response times, indicating that negative valence affects processing speed similarly across age groups. This suggests that the influence of negative valence on processing speed might be a general effect rather than one exacerbated by ageing. However, the allowance of a long response window adapted in our study may also have contributed to these findings.

Differences in functional connectivity between young adults and old adults during retrieval were consistently observed following exposure to negative valence, with connectivity changes being prominent in the right cuneus, left lingual gyrus, and left inferior occipital gyrus. These regions are associated with visual processing, suggesting that the observed connectivity changes following exposure to negative valence may reflect alterations in retrieval mechanisms in ageing adults. We also observed disparities in the right inferior parietal gyrus—part of the default mode network (DMN)—and the middle frontal gyrus, both implicated in attention processes [100]. Notably, while previous studies reported activity in the right middle frontal gyrus [101], our findings indicate activity in the left hemisphere of the same region. These differences were notable again following negative valence, indicating that the ageing brain employs these regions differently, especially in response to negative emotional stimuli. The altered pattern of connectivity could be a response to the decreased efficiency in the engagement of neural pathways typically utilised by young adults [102]. Up to that point, we faced ambiguity regarding the directionality of observed functional connectivity differences, as it remained uncertain whether these variations suggest heightened connectivity in young compared to old adults or decreased coupling following exposure to negative valence. Consequently, we examined the functional connections associated with these regions to shed light on potential compensatory adaptations within the ageing cohort. Given the significant influence of negative valence on the behavioural findings and its persistent role in elucidating age-related neural differences, we further identified how these regions connect to the rest of the brain, bridging the gap between the behavioural and neural findings.

Our refined focus initially revealed age-related disparities in the functional coupling of the identified seed regions with the rest of the brain. Specifically, we observed heightened connectivity in young adults, compared to old adults. In young adults, the right inferior parietal gyrus displayed increased connectivity with the right precuneus and lingual gyrus, suggesting a network linking attentional processes with visual perception [103] and episodic memory processes. Furthermore, the left anterior insula, implicated in emotional awareness and interoceptive experiences [104], emerged as a central hub exhibiting connections with the left precentral gyrus, right superior medial frontal gyrus, left lingual gyrus, and left fusiform gyrus. This connectivity pattern implies the use of a network by young adults involved in the regulation of emotional processes [105] and its integration of sensory–motor information. Additionally, we observed coordination between the left middle frontal gyrus and the left paracentral lobule, indicating collaboration between attentional processes and motor planning. These connections offer insights into the superior memory performance observed in young adults compared to old adults, as they suggest a more efficient functional integration in the former group.

The older adult group, in comparison to young adults, displayed an increase in functional connections on only two occasions, and notably, both occurred following exposure to positive valence. Specifically, the observed coupling between the right cuneus and the right precentral gyrus suggests a coordinated interaction between visual processing and motor intention, respectively. This might reflect older adults’ utilisation of alternative neural pathways to support memory retrieval processes. Similarly, the enhanced coupling between the left anterior insula and the left inferior occipital gyrus in response to positive valence implies a facilitation of adaptive responses to positive stimuli prompting the integration of emotional and visual processes. These connectivity patterns potentially contributing to better memory performance under positive conditions compared to negative conditions which aligns with our behavioural findings. Our findings point to the possibility that positive valence may facilitate existing connections in the older adult brain, perhaps as a compensatory mechanism [9,35]. Such enhancement prompts the utilisation of these neural strategies during memory retrieval, aiding in maintaining functionality despite age-related decreases in functional connectivity.

While old adults displayed increased functional connectivity only in response to positive valence, results highlighted a persistent trend of heightened connectivity following exposure to negative valence in young adults. Specifically, we noted increased coupling between the right inferior parietal gyrus and the precuneus, as well as between the left middle frontal gyrus and the paracentral gyrus. Heightened connectivity between the right inferior parietal gyrus and the precuneus might be associated with an intensified effort to process and integrate sensory information presented following negative valence exposure, managing its influence. Similarly, heightened connectivity between the left middle frontal gyrus, associated with working memory and attention [106], and the paracentral gyrus reflects increased effort and motor planning following negative valence. This potentially suggests deliberate decision-making or heightened vigilance in response to negative stimuli. Our observations imply that the healthy young adult brain mobilises additional resources to address challenges posed by negative stimuli to potentially mitigate the impact of negative valence on task performance. The heightened connectivity observed in response to negative valence aligns with previous findings supporting the influence of negative valence on episodic memory [39,40,41,42]. Additionally, these results are consistent with the Dynamic Integration Theory [107], which suggests an advantage for positive stimuli because they require fewer cognitive resources, leading to less effortful strategies, which, in our study, is reflected as decreased functional connectivity following exposure to positive valence.

In our study, the imbalance in sample sizes between the two groups posed a potential limitation. To address this challenge and mitigate its impact on our findings, we employed mixed modelling, which is particularly effective at handling variations in group sizes, ensuring that our analyses remain robust despite the disparity [88]. Another challenge encountered in our study design was the repetition of videos which could raise concerns about potential familiarisation effects. To rigorously address this issue, we incorporated video repetition as a fixed factor in our mixed modelling analysis. This statistical adjustment was a critical component of our methodology, designed to systematically control for and negate any confounding influences arising from repeated video exposure. By employing mixed modelling, we aimed to effectively address the inherent limitations of our study design and sample size disparities, thereby enhancing the reliability and validity of our findings.

Moreover, it is important to highlight several factors that complicate the direct comparison between positive and negative stimuli. Negative stimuli generally provoke higher arousal levels than positive stimuli [108], as an image of an injured or deceased person is inherently more distressing than a picture of a mother hugging her baby. Additionally, although we did not collect individual valence ratings form our subjects, it is well established that individual perception of positive and negative affects varies [109]. There is also growing evidence of a positivity bias, where people process positive information faster and form broader associations from it [110]. Furthermore, standardising emotionally valenced complex information, such as human faces or images of emotional scenes, poses methodological challenges [111]. Recognising these complexities, we suggest that future research consider controlling for arousal to provide a clearer understanding of emotional stimuli processing. In the future, it may be a good direction for research to use eye tracking with saliency maps, which are designed to predict consecutive fixation locations [112]. This approach could provide more detailed information about attention allocation and improve our understanding of how the brain processes different emotional stimuli.

Moving forward, we suggest a shift toward examining functional connectivity to explore age-related changes comprehensively. This study presents our initial effort to analyse ageing theories through the lens of functional coupling, aiming for a more holistic understanding of neural activity. Functional connectivity measures proved invaluable in our study as they allowed for the investigation of multiple processes simultaneously, in our case episodic memory and the impact of emotional valence. Given that ageing is associated with both structural and functional changes, examining functional connections provides an integrated perspective. We believe that future research should focus on these aspects to advance our understanding of the ageing brain.

Our findings indicate that the ageing brain remains susceptible to the disruptive effects of negative valence on subsequent memory retrieval processes. Our data-driven approach allowed for a comprehensive analysis of functional connections, enhancing our understanding of memory processes following exposure to emotional stimuli. While young adults utilised extensive functional networks, old adults employed a more focused strategy enhancing specific connections to potentially compensate for declines in functional connectivity. We suggest that while negative valence in young adults prompts increased functional connectivity, reflecting an adaptive response in mitigating its impact on task performance, positive valence can facilitate connectivity in old adults. This underscores the importance of considering emotional processes in memory retrieval disruptions and improving cognitive interventions for old adults.

## Figures and Tables

**Table 1:** MVPA results of the specific seeds displayed age-related variations in functional connectivity during episodic memory retrieval following valence exposure.

	H	Peak Region	MNI Coordinates			Voxel No.	BSR
Young > Old			x	y	z		
	R	Cuneus	+10	−78	+32	641	0.000000
	L	Middle Frontal Gyrus	−28	+58	+12	109	0.000000
	L	Lingual Gyrus	−18	−84	−16	98	0.000001
	L	Inferior Occipital Gyrus	−54	−72	−2	79	0.000010
	L	Anterior Insular Cortex	−28	+14	+12	76	0.000010
	R	Inferior Parietal Gyrus	+38	−52	+42	70	0.000040

Abbreviations: BSR = bootstrap ratio; H = hemisphere; L = left; R = right; Voxel No. = Number of Voxels; x coordinate = right/left; y coordinate = anterior/posterior; z coordinate = superior/inferior.

**Table 2:** Results from the second SBC using semipartial correlations displaying age-related differences in functional connectivity between seed regions derived from the MVPA and the rest of the brain during episodic memory retrieval across valence conditions.

Group Contrast	Condition Contrast	H	Peak Region	MNI Coordinates			Voxel No.	BSR
				x	y	z		
**Right Cuneus**								
old > young	pos > neu	R	Precentral Gyrus	+60	+8	+20	170	0.0005
**Right Inferior Parietal Gyrus**								
young > old	neg > neu	R	Precuneus	+6	−62	+66	172	0.0003
young > old	pos > neu	R	Lingual Gyrus	+22	−84	−12	144	0.0015
young > old	neg > pos	R	Precuneus	+8	−62	+66	124	0.0038
**Left Anterior Insula**								
young > old	neg > neu	L	Precentral Gyrus	−40	+8	+36	158	0.0005
old > young	pos > neu	L	Inferior Occipital Gyrus	−36	−72	−10	181	0.0002
young > old		R	Superior Medial Frontal Gyrus	+12	+56	+14	176	0.0002
young > old	neg > pos	L	Lingual Gyrus	−2	−78	+4	143	0.0015
young > old		L	Fusiform Gyrus	−34	−74	−12	123	0.0047
**Left Middle Frontal Gyrus**								
young > old	neg > pos	L	Paracentral Lobule	−4	−22	+70	191	0.0001

Abbreviations: BSR = bootstrap ratio; H = hemisphere; L = left; R = right; Voxel No. = Number of Voxels; x coordinate = right/left; y coordinate = anterior/posterior; z coordinate = superior/inferior.

**Figure A1:**
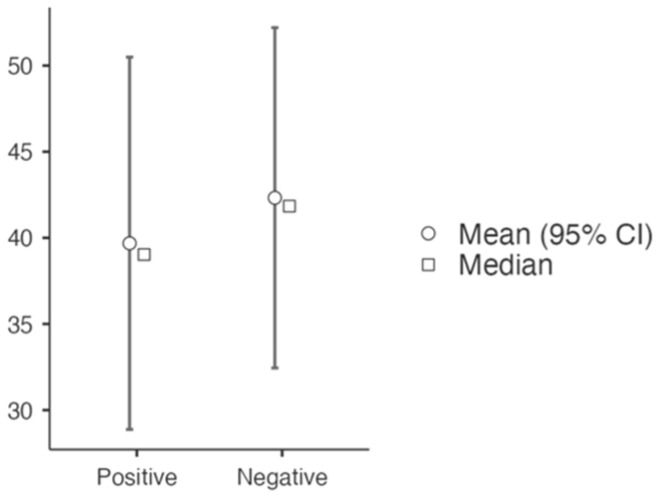
Mean scores of arousal ratings for positive and negative stimuli.

**Figure A2:**
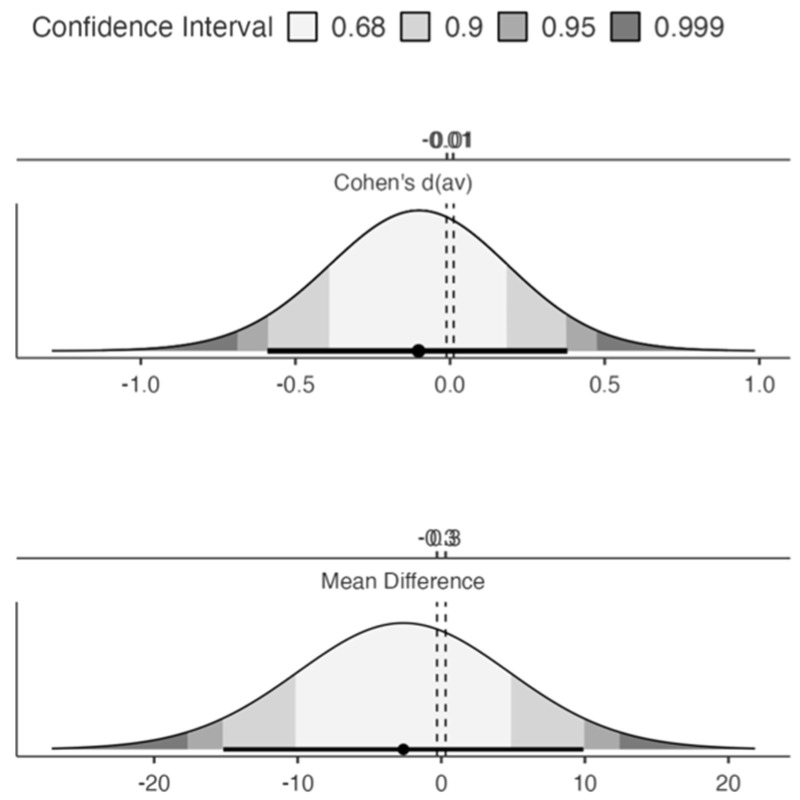
Equivalence test of arousal ratings of positive and negative stimuli.

## Data Availability

The data presented in this study are available on request from the corresponding author due to privacy and ethical reasons.

## Appendix A

We tested the difference between arousal scores for positive and negative images using an independent samples *t*-test. Following recent recommendation to use more robust procedures of testing the effects of the differences between two independent groups [113,114,115], we also used the equivalence test implemented in the TOSTER package in Jamovi (version 2.5.7.0).

Prior entering the data into the analyses, the arousal ratings for positive and negative images were standardised. An independent sample *t*-test showed that the mean difference between the ratings were non-significant (t(46) = −0.35, *p* = 0.73, BF_10_ = 0.30) (Figure A1).

For the equivalence test, we selected the smallest effect size corresponding to Cohen’s d = 0.3 (with the lower bond = −0.3 and the upper bond = 0.3). By rejecting an effect more extreme that these pre-defined equivalence bonds, we can conclude that the true effect is close enough to zero for the practical purpose [116]. This approach allows us to provide a robust testing the equivalence using a relatively small sample on our picture (24 pictures per a condition). The equivalence test did not reject the null hypothesis (upper bound: t(45.63) = −0.31, *p* = 0.62; lower bound: t(45.63) = −0.39, *p* = 0.35) (Figure A2).

## Appendix B

1. Accuracy

Analysis of the accuracy data, employing a fixed-effect omnibus test, identified a main effect of Age (χ^2^(1) = 7.70, *p* = 0.006), showing enhanced accuracy in young adults compared to old participants (SE = 0.08, z = 2.77). A main effect of Valence is also reported (χ^2^(2) = 13.29, *p* = 0.001), with further examination through post-hoc evaluations, adjusted for multiple comparisons using the Bonferroni method, revealing significantly lower accuracy rates in the Negative versus Neutral condition (MD = 0.691, SE = 0.072, z = −3.57, p_bonf_ < 0.001). The MDs between the two valenced conditions and between the Neutral and Positive conditions were not significant (MD = 0.815, SE = 0.0827, z = −2.02, p_bonf_ = 0.131, and MD = 1.180, SE = 0.125, z = 1.56 p_bonf_ = 0.353 respectively). No significant interaction between Age and Valence was observed (χ^2^(2) = 2.50, *p* = 0.287). The variability attributed to the random effects was minor (Participants: SD = 0.373, σ^2^i = 0.139, CI [0.0678, 0.262], ICC = 0.041, and Video: SD = 0.481, σ^2^i = 0.231, CI [0.1203, 0.470], ICC = 0.0656).

2. Response times

Analysis of the response time data, employing a fixed-effect omnibus test, did not identify a main effect of Age (F(1, 2784) = 0.0717 *p* = 0.789). No significant interaction between Age and Valence was observed (F(2, 2784) = 0.3183, *p* = 0.727). A main effect of Valence is reported (F(2, 2790) = 47.05 *p* < 0.001), with further examination through post-hoc evaluations, adjusted for multiple comparisons using the Bonferroni method, revealing significantly slower responses in the Negative compared to the Neutral condition (MD = 0.343, SE = 0.0402, t(2754) = 8.55, *p*_bonf_ < 0.001) and between the two arousing conditions (MD = 0.337, SE = 0.0404, t(2760) = 8.35, *p*_bonf_ < 0.001). No significant difference is reported between the Neutral and Positive condition (MD = −0.0058, SE = 0.0395, t(2763) = −0.147, *p*_bonf_ = 1.00). The variability attributed to the random effects was minor (Participants: SD = 9.42 × 10^−9^ and Video: SD = 0.279).

## Appendix C

### Seed-Based Connectivity (SBC)

For young adults compared to old adults, in the negative versus neutral condition, enhanced functional connectivity was found between the left inferior occipital gyrus and the right parahippocampal gyrus, along with regions within the left occipital lobe. In the positive versus neutral condition, an increased functional connection was observed with the right calcarine. For the negative versus positive condition, the left inferior occipital gyrus showed stronger connectivity with the right superior frontal gyrus and left angular gyrus. Moreover, the left anterior insula in young adults exhibited greater functional connectivity with the frontal gyrus bilaterally across both valenced conditions compared to the neutral condition. Additionally, the left middle frontal gyrus displayed more pronounced connectivity with the left parietal and right fusiform gyrus during the negative versus neutral condition, and increased connectivity with the right lingual gyrus and left insula for the negative compared to positive condition. Finally, the right inferior parietal gyrus was functionally connected to the right superior parietal and left precuneus for the negative compared to positive condition.

On the other hand, in old adults compared to young adults, the right cuneus was functionally connected more to regions in the occipital gyri and right superior temporal gyrus for the negative comparted to neutral condition, while the negative compared to positive condition displayed increased connections to the right calcarine and right lingual gyrus. The left lingual gyrus displayed increased functional connectivity to the occipital cortex for the negative compared to neutral condition, the left inferior parietal gyrus for the positive compared to neutral and finally regions in the left frontal and superior occipital gyrus for the negative compared to positive condition. Lastly, the right inferior parietal gyrus in old adults was found to be more functionally connected to the left temporal gyrus during the negative compared to neutral condition.

Table A1 displays all significant functional connections with clusters at *p*-FWE > 0.005 in young adults compared to old adults and old adults compared to young adults respectively.

**Table A1:** Results from the first SBC showing age-related differences in functional connectivity between seed regions derived from the MVPA and the rest of the brain during episodic memory retrieval across valence conditions.

		H	Peak Region	MNI Coordinates			Voxel No.	BSR
	Contrast			x	y	z		
1. Right Cuneus								
old > young	neg > neu	R	Superior Occipital Gyrus	+18	−90	+18	3495	0.0000
old > young		L	Lingual Gyrus	−8	−64	−4	1102	0.0000
old > young		L	Superior Temporal Gyrus	+62	−12	+2	168	0.0008
old > young	pos > neu	R	Calcarine	+16	−100	+2	152	0.0016
old > young	neg > pos	R	Calcarine	+8	−94	+12	167	0.0006
old > young		R	Lingual Gyrus	+14	−42	−6	84	0.0706
2. Left Lingual Gyrus								
old > young	neg > neu	R	Precuneus	+18	−68	+40	1062	0.0000
old > young		L	Superior Occipital Gyrus	−14	−86	+32	324	0.0000
old > young		L	Middle Occipital Gyrus	−30	−70	+42	254	0.0000
old > young		R	Middle Occipital Gyrus	+44	−68	+26	178	0.0003
old > young	pos > neu	L	Inferior Parietal Gyrus	−30	−58	+42	236	0.0000
young > old	neg > pos	L	Inferior Tri Frontal Gyrus	−44	+32	+8	134	0.0032
old > young		L	Superior Occipital Gyrus	−10	−98	+22	128	0.0044
3. Left Inferior Occipital Gyrus								
young > old	neg > neu	R	Parahippocampal Gyrus	+24	−40	−10	176	0.0003
young > old		L	Fusiform Gyrus	−32	−38	−24	174	0.0004
young > old		L	Lingual Gyrus	−12	−50	+4	167	0.0005
young > old		L	Cuneus	−4	−76	+36	163	0.0007
young > old	pos > neu	R	Calcarine	+16	−58	+6	309	0.0000
young > old	neg > pos	R	Superior Frontal Gyrus	+16	+66	+2	259	0.0000
young > old		L	Angular Gyrus	−50	−60	+42	139	0.0027
4. Right Inferior Parietal Gyrus								
old > young	neg > neu	L	Superior Temporal Gyrus	−48	−26	+4	480	0.0000
old > young		L	Superior Temporal Pole	−44	+20	−14	132	0.0043
young > old	neg > pos	R	Superior Parietal	+12	−56	+72	173	0.0005
young > old		L	Precuneus	−14	−62	+64	132	0.0042
5. Left Anterior Insula								
young > old	neg > neu	L	Superior Medial Frontal Gyrus	−8	+50	+36	1567	0.0000
young > old		L	Inferior Tri Frontal Gyrus	−36	+16	+30	538	0.0000
young > old		L	Superior Frontal Gyrus	−34	+52	+2	202	0.0001
young > old		R	Middle Frontal Gyrus	+32	+58	+6	157	0.0009
young > old	pos > neu	L	Superior Medial Frontal Gyrus	−2	+50	+30	1092	0.0000
young > old		R	Middle Frontal Gyrus	+46	+32	+24	168	0.0005
6. Left Middle Frontal Gyrus								
young > old	neg > neu	L	Superior Parietal Gyrus	−24	−56	+42	186	0.0002
young > old		R	Fusiform Gyrus	+30	−74	−6	162	0.0007
young > old	neg > pos	R	Lingual Gyrus	+20	−70	−6	243	0.0000
young > old		L	Insula	−44	0	−6	177	0.0003

Abbreviations: BSR = bootstrap ratio; H = hemisphere; L = left; R = right; Voxel No. = Number of Voxels; x coordinate = right/left; y coordinate = anterior/posterior; z coordinate = superior/inferior.
